# One potential method of recycling expired thiocolchicoside drug is to use an environmental corrosion inhibitor for carbon steel in HCl

**DOI:** 10.1038/s41598-025-07609-y

**Published:** 2025-07-30

**Authors:** Mariem M. Motawea, Ayman A. O. Younes, Islam Ragab, Amira A. E. Satti, Rabeea M. A. Daoub, Seham H. Bashir

**Affiliations:** 1 Department of Chemistry, College of Science, University of Bisha, P.O. Box. 551, 61922 Bisha, Saudi Arabia; 2 Department of Chemistry, College of Science, Qassim University, Buraidah 51452, Saudi Arabia; 3 Department of Chemistry, College of Science, Jouf University, Sakaka, Aljouf 72341, Saudi Arabia; 4 Department of Chemistry, College of Science, Northern Border University, Arar, Saudi Arabia; 5 Chemistry Department, Faculty of Science, Al-Baha University, Al-Baha, Kingdom of Saudi Arabia; 6 Department of Chemistry and Industrial Chemistry, College of Applied and Industrial Sciences, University of Bahri, Khartoum, Sudan

**Keywords:** 1040Carbon steel, Corrosion Inhibition, AFM, FTIR, Thiocolchicoside drug, Electrochemistry, Green chemistry, Materials chemistry

## Abstract

One of the problems facing our society today is managing hazardous waste. Expired pharmaceuticals are a type of waste that is not recycled in any way but is instead burned for disposal. These pharmaceutical drugs have been shown to have corrosion-inhibiting properties for various metals and corrosive solutions. The current study examined the corrosion tendency of 1040 carbon steel (1040CS) under acidic pH (1 M HCl) by using an unused, expired Thiocolchicoside (ETC) drug as a possible corrosion inhibitor was studied by using the weight loss (WL), electrochemical impedance spectroscopy (EIS), and potentiodynamic polarisation (PP) tests, and. It was observed that the increase in ETC doses led to the rise in corrosion IE (%) up to 93.3, 90.7, and 91.4%, as shown by EIS, PDP, and WL techniques, respectively. However, inhibition efficiency diminished with rising temperatures, declining from 91.83% at 298 K to 78.63% at 318 K (all at 400 ppm). The results obtained high Rct during electrochemical processes, which reduced Icorr and, in turn, the ETC molecule strong adsorption onto the 1040CS surface. The PP data showed that this ETC decreased the corrosion current density by a mixed-type mechanism. SEM, AFM, and FTIR surface studies demonstrated the development of a protective layer at the CS surface. DFT-based quantum chemical indices made further understanding of the inhibitory mechanism possible. Results gained for all techniques used are in good agreement.

## Introduction

Carbon steel is the preferred structural material because it is inexpensive, readily available, and has excellent mechanical properties but it corrodes both chemically and electrochemically quickly. Acid solutions act as corrosive media and have a significant role in industries that eliminate corrosion products through the petrochemical process, acid cleaning, acid descaling, acid pickling, and accelerating corrosion. Also, HCl is an example of acid solution^1,2^. Carbon steel corrodes as a result of chemical reactions with the environment. This process is influenced by oxidation, moisture, acids, impurities, and electrical stress, among other things. Thus, corrosion is one of the most expensive and risky processes that endangers all sectors. It may cause buildings and bridges to collapse, oil pipelines to burst, chemical facilities to leak, etc^3^. One way to think of corrosion is as a substance organic breakdown brought on by an unintentional interaction with its surroundings^4^. Corrosion arises from electrochemical reactions where metal surface atoms relinquish electrons to an electron acceptor (like oxygen, acids, or cations of less active metals) present in air or aqueous solutions. This oxidation process deteriorates the entire metal surface^5–7^. The use of organic corrosion inhibitors to prevent acid corrosion of C-steel is commonly overused. The majority of past research on the use of heterocyclic inhibitors to control acid corrosion of C-steel has focused on the inhibitor’s effectiveness. Nonetheless, there aren’t many research on how performance varies depending on which substituents are present in a given heterocyclic nucleus. Thus, a study employing pharmaceutical compounds was conducted to investigate the corrosion inhibition of C-steel in 1 M HCl media^8^.

Numerous studies have supported using pharmaceuticals to prevent corrosion as an environmentally responsible method with no adverse effects^9^. Pharmaceutical substances can act as antagonists to green corrosion inhibitors^10^. With the help of plentiful natural resources, most items can be synthesized. Carbocyclic or heterocyclic systems were widespread in the field of drug structures. Numerous considerations influenced the choice of the best pharmaceutical agent for corrosion mitigation^11–14^. The presence of sulfur, oxygen, and nitrogen within drug molecules is critical for effective corrosion mitigation. Identifying an optimal pharmaceutical inhibitor can be facilitated by considering two key parameters: the compound’s molecular dimensions and solubility characteristics^15^.

Heterocyclic compounds such as antibiotics (pharmaceutical drugs) can provide excellent inhibition^16–18^. Several studies have highlighted the suitability of antibacterial drugs for corrosion inhibition. These drugs are attributed to active centers like oxygen, nitrogen, and sulfur in their structures, their high water solubility, substantial molecular size, non-toxicity, and environmental friendliness. Moreover, these molecules are often involved in crucial biological processes and are readily produced and purified, making them attractive candidates for corrosion inhibition applications^19–25^.

Utilizing waste medications can face two significant environmental and economic issues: limiting environmental contamination with pharmaceutically active chemicals and lowering the expense of disposing expired drugs^26^. On the other side, unwanted medicines are often neutralized by burning, which risks contaminating the environment with hazardous chemicals, including N, S, P, or halogen atoms. Recently, we have examined the inhibiting action of Pharmaceutical drugs (Table 1).

**Table 1:** Inhibition efficiency of pharmaceutical drug inhibitor for metal corrosion.

Metal	Media	IE %	Inhibitor drug [Ref.]
C-steel	1 M HCl	78.3%	Phenobarbitone^27^
C-steel	0.5M H_2_SO_4_	82.2%	Glucosamine^28^
C-steel	1 M HCl	79.8%	Candesartan^29^
Mild steel	1 M HCl	61.1%	Meloxicam^30^
C-steel	0.5 M H_3_PO_4_	80.6%	Expired etoricoxib^31^
C-steel	1 M HCl	87.3%	Aminophylline^32^
Mild steel	1 M HCl	67.5%	Cefalexi^33^
C-steel	1 M HCl	82%	Tetracycline drug^34^
C-steel	1 M HCl	86%	Paracetamol^35^
Mild steel	1 M HCl	70.4%	Expired ambroxol^36^
Mild steel	1M H_2_SO_4_	76%	Flucloxacillin^37^
C-steel	3M H_3_PO_4_	69.3%	lansoprazole^38^

As a result, our research focused on using unused medicines due to patient failure, including active compounds with inhibitory characteristics in their composition. Because of their economic benefits, high corrosion inhibitor efficiency, and environmental friendliness, they make an excellent substitute for the industry’s synthetic inhibitors. The research target of this study was New ETC, where severe corrosion effects on tools/farm equipment and implements produced from ETC/Fe were observed. It is believed that this research may promote the utilization of ETC for the protection behavior of 1018CS in 1 M HCl to solve these corrosion effects. The surface morphology of the 1018CS specimens is evaluated.

## Experimental techniques

### Materials

The chemical composition of 1040CS (in weight%) is displayed in (Table 2). The exposed metal surface area for electrochemical experiments was 1 cm^2^while the exposed surface area of a 1040CS specimen, measuring 2 × 2 × 0.2 cm, was utilized for chemical studies. The surface of 1040CS specimens was mirror-finished using the best emery paper before each experiment, rinsed with acetone, and then washed with triple-distilled water.

**Table 2:** Chemical conformation (weight%) of the 1040 Steel.

Element	C	Si	Mn	*P*	S	Fe
%	0.4	0.003	0.75%	< 0.04%	< 0.05%	the rest

### Hydrochloric acid solution

1 M HCl, a corrosive medium, was created by diluting a sufficient 37% concentrated chemically pure grade acid using distilled water. Titration of an appropriately diluted amount against standard sodium carbonate solution was used to determine the acid’s concentration.

### Inhibitor and chemicals

**Fig. 1:**
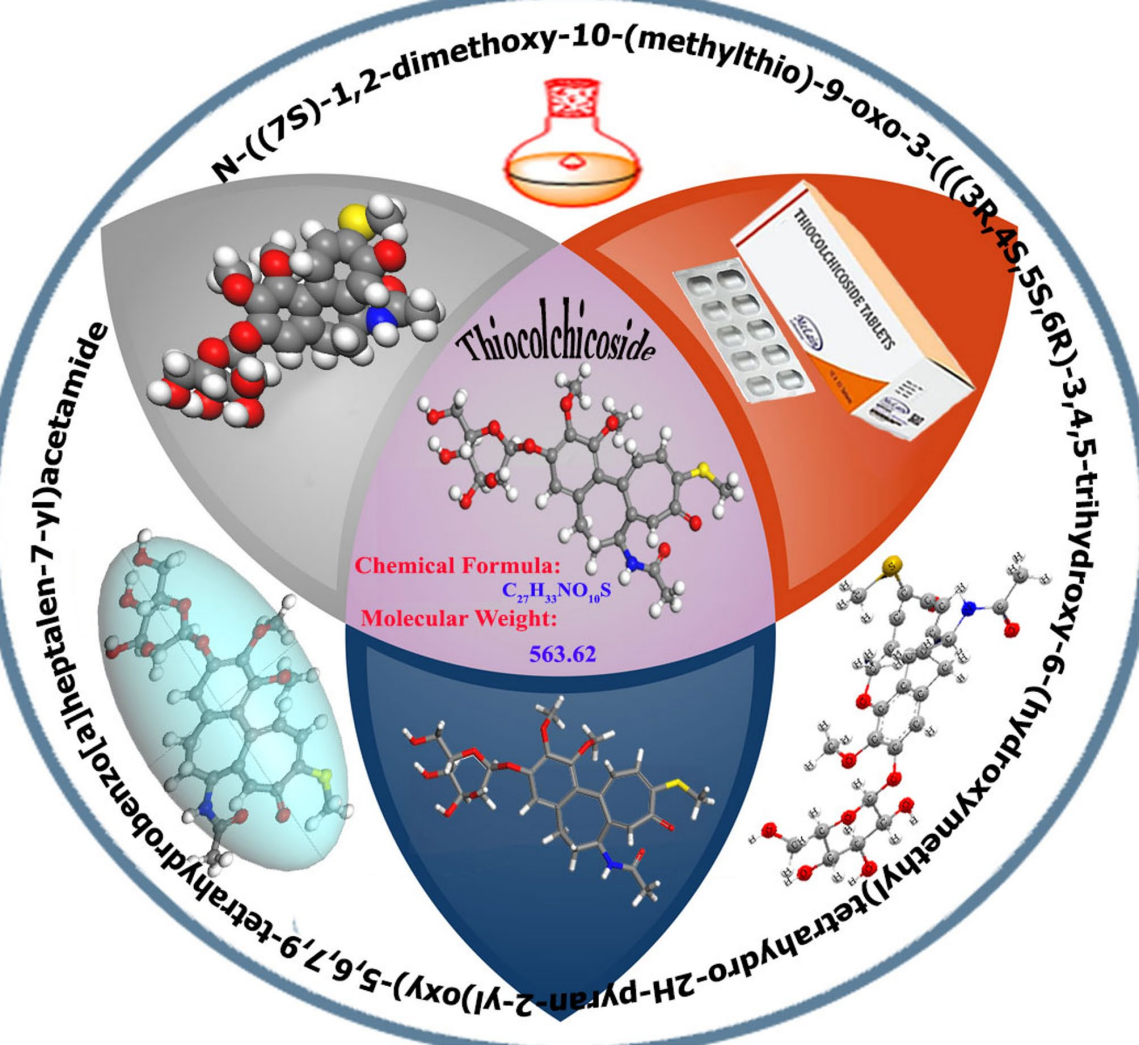
Molecular structure, formula, and weight of investigated drug (Microsoft 365PowerPoint, https://www.microsoft.com/en-us/microsoft-365/powerpoint).

The pharmaceutical compound, an unused expired Thiocolchicoside (ETC) drug, has been examined (Fig. 1). One gram per liter of the solid pharmaceutical drug was dissolved in bi-distilled water to create 1000 ppm stock solutions from the investigated drug; the remaining doses of the pharmaceutical drug (50–300 ppm) were prepared by dilution with bi-distilled water. The investigated pharmaceutical drug is used as received and chosen because it is easily soluble in water, has a high molecular weight, and contains many donating atoms (N, O, and S). All the solutions were prepared from AR-grade chemicals using bi-distilled water.

### Methods

#### Chemical measurements (WL) measurements

Measurements of weight loss corrosion were performed in accordance with ASTM protocol^39^. A common and better technique used in corrosion laboratories to evaluate how well inhibitors reduce corrosion resistance is WL testing. After sanding the C-steel coupons with varying grit levels (P220–P2000 ISO/FEPA Grit) until a mirror surface was obvious, the coupons were rinsed with bidistilled water and allowed to air dry at room temperature. The metal coupons were weighed before and after immersion in 100 ml of 1 M hydrochloric acid solutions containing various concentrations of the ETC or no ETC. This process was repeated every 30 min for a total of 3 h. At 298, 303, 308, 313, and 318 K, among various temperatures, the study was carried out. A weight loss method was used to evaluate corrosion rate (k_corr_), corrosion inhibition efficiency (% IE), and surface coverage ($\:{\uptheta\:})$ by the following balance^40^.

1 $$\% {\text{ }}IE{\text{ }}={\text{ }}\Theta {\text{ }} \times {\text{ }}100{\text{ }}={\text{ }}\left[ {1 - {\text{ }}\left( {{W_1}/{W_2}} \right)} \right]{\text{ }} \times {\text{ }}100$$

2 $${k_{corr}}={\text{ }}W/{\text{ }}At$$

where W_1_ and W_2_ are the WLs for 1040CS in the attendance and lack of the drug, A is the area of the 1040CS coupon (cm^2^), t is the time of immersion (min), and W is the WL (mg cm^−2^) of 1040CS after time t (min).

#### Electrochemical measurements

A three-electrode electrochemical cell was used. The working electrode was 1040CS with a surface area of 1 cm^2^. Before each experiment, the electrode was abraded using emery papers as before. Using a potentiostate/galvanostate (Gamry PCI 300/4) and a personal computer running EIS 300 software. After this, the electrode was cleaned ultrasonically with ethyl alcohol and washed with bi-distilled water. All potentials regarding the saturated calomel electrode (SCE) were given. The counter electrode was a platinum plate with a surface area of 1 cm^2^. The working electrode was immersed in the test solution for 30 min until a steady state open circuit potential (E_ocp_) was obtained. All tests took place in stagnant solutions, as in WL measurements.

In the case of 10040CS, the electrode potential was automatically changed from − 1.2 to 0.4 V with a scanning rate of 1 mV s^− 1^ to record the potentiodynamic current-potential curves^41^. All experiments were conducted at 25 ℃ without de-aerating the solutions using an ultra-circulating thermostat. All experiments were carried out at 25 °C. The %IE and (θ) were calculated from the following equation:

3 $$\% IE = \Theta \times 100 = [1 - \left( {{i_{corr(inh)}}/{i_{corr(free)}}} \right] \times 100$$

where, i_corr(free)_ and i_corr(inh)_ are the corrosion current densities in the absence and presence of an inhibitor, respectively.

EIS measurements were carried out. The electrode was submerged for 30 min before the EIS spectra were recorded at open circuit potential (OCP). An EIS diagram was obtained from 1 Hz to 100 kHz using ten points per degree and a 10 mV rms AC signal. Calculating an anticorrosion efficiency value requires fitting an electrical equivalent circuit using the following equation:

4 $$\% {\text{ }}IE{\text{ }}={\text{ }}\Theta {\text{ }} \times {\text{ }}100{\text{ }}={\text{ }}[1 - {\text{ }}\left( {R{^\circ _{ct}}/{\text{ }}{R_{ct}}} \right] \times {\text{ }}100$$

where Rº_ct_ and R_ct_ are the resistance data in the lack and attendance of the inhibitor, correspondingly.

### Surface examinations^42^

#### Atomic force microscopy (AFM) analysis

The AFM technique was used to determine the morphological characteristics of the 1040CS surface. The test is conducted in 1 M HCl when expired drug inhibitors are absent, and their maximum dosage is present. The analysis was performed using atomic force microscopy (AFM) on a Pico SPM2100 AFM device operating in contact mode in the air.

#### Scanning electron microscopy (SEM)

Scanning electron microscopy (SEM) using a JEOL-JSM-6510LV model in Japan was used to examine the surface morphology and elemental composition of glossy and corroded 1040CS samples, both with and without the inhibitor. It was used to confirm that an inhibitory surface coating had formed at the electrode surface and, hence, caused corrosion inhibition.

#### Fourier transform infrared spectroscopy technique (FT-IR)

FT-IR measurements provide details about the function groups in the studied inhibitors. The composition of the corrosion product generated on the 1040CS surface was ascertained by recording the FTIR spectra using an IR Affinity (PerkinElmer) spectrophotometer. The CS sample surface submerged in the free acid solution as well as an inhibited solution using the examined drug inhibitors.

### Quantum chemical calculations

Theoretical calculations were performed using the DMol3 module installed in Materials Studio model 7.0 to observe the correlation between the molecular structure and the reactivity of Unused ETC drug. The Frontier orbital theory reports that HOMO and LUMO energies control the interaction between the inhibitor molecule and the metal^43^. Whereas E_LUMO_ measures the molecule’s ability to accept electrons, E_HOMO_ measures the molecule’s ability to donate electrons. The quantum chemical parameters (E_LUMO_, E_HOMO_ and $\:\varDelta\:$E) have a strong relationship with the protection efficiency of the investigated drug. As a result, an inhibitor molecule with low E_LUMO_ and high E_HOMO_ values exhibits increased corrosion inhibition efficacy.

### Monte Carlo (MC) simulations

Materials Studio 7.0 (Accelrys Inc., San Diego, CA, USA) created MC simulations in a simulation box with recurring boundary conditions. The inhibitor’s conformational adsorption modification on the iron surface was also predicted using Monte Carlo simulations. To create a 30 vacuum slab, a pure iron crystal was added and cleaved along the most stable plane (had the least energy, 1 1 0). The plane surface of Fe (1 1 0) has been relaxed by decreasing its energy; this step has been followed by extending the surface of Fe (1 1 0) to a supercell (10/10). A Monte Carlo simulation of the ETC drug on Fe (110) was conducted using the COMPASS force field in Materials Studio 7.0 for the aqueous phase. Simulation techniques are widely used in corrosion inhibition research, enabling investigation without hazardous chemicals or lab equipment. This approach explores the link between molecular structure and simulated outcomes, advancing corrosion inhibitors^44^.

## Results and discussion

### WL measurements

**Fig. 2:**
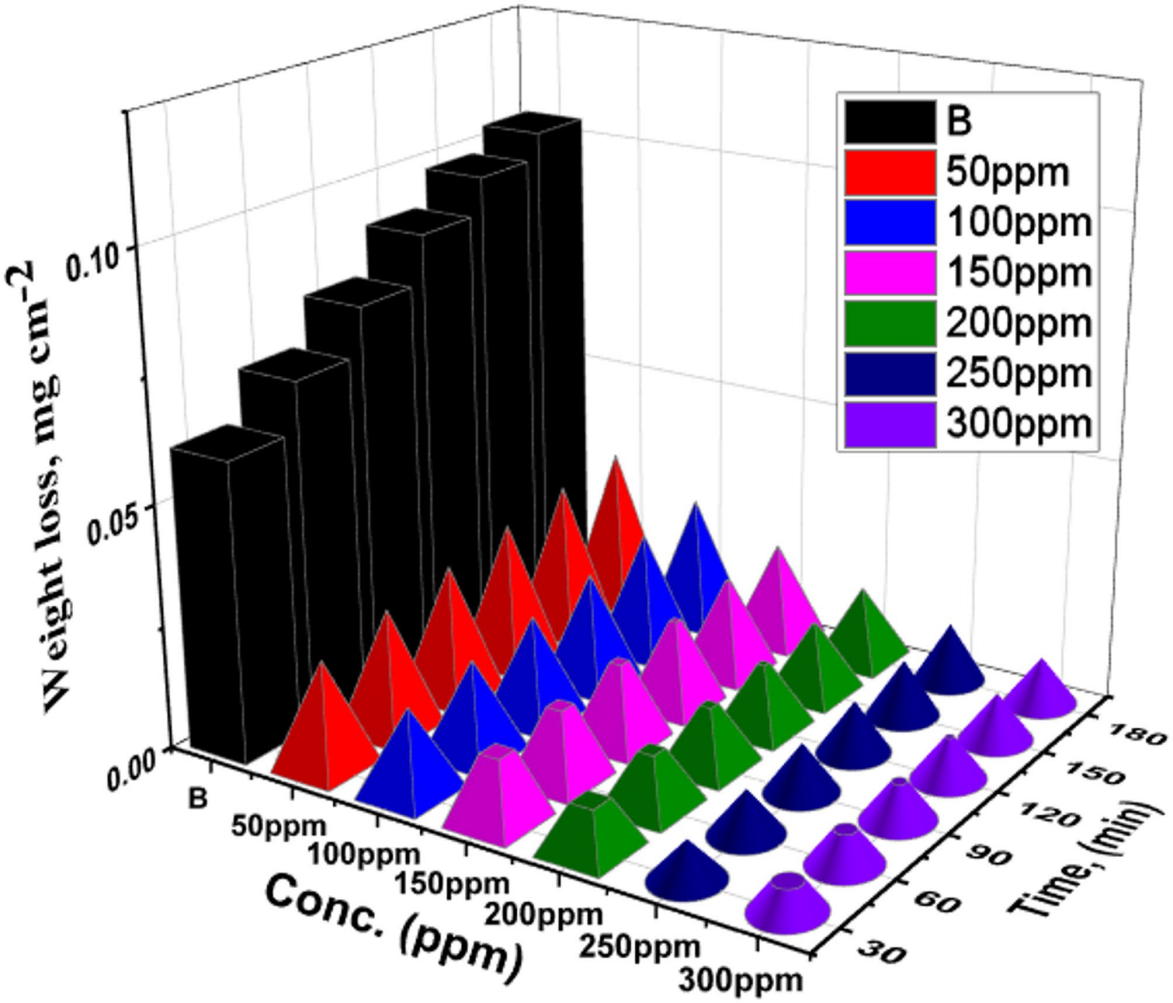
Time-WL plots for the 1040CS dissolution in 1 M HCl solution in the absence and attendance of altered doses of Unused ETC drug at 25 °C.

For weight loss analysis, CS samples were produced in accordance with ASTM G 31–72^45^. The inhibition efficiency (% IE) rises, and the corrosion rate (k_corr_) falls as the dose of ETC is increased to an optimal level of 300 ppm. Subsequent increases in ETC dose do not result in substantial changes in % IE or k_corr_. ETC achieved a maximum IE of 91.4% in HCl at the optimal dose of 300 ppm. This result could be explained by increasing 1040CS surface area coverage by the ETC molecule. At the maximum dose, the corrosion rate, as measured by weight loss, is lowest, while the inhibition efficiency is highest. The % IE rises with improving drug doses and lowers with a rise in temperature. Further increments in ETC drug dose did not yield significant changes in the drug performance. There’s also the potential for a vigorous interaction between the ETC attached to the metal surface and the molecular drug in the solution^46,47^. According to these findings, the understudied ETC medication effectively inhibits 1040CS dissolution in 1 M HCl solution. The findings demonstrated that the Unused ETC medication positively impacted the corrosion inhibition of 1040CS in a 1 M HCl solution, which is a corrosive medium. Figure 2 shows the WL-time curves for the corrosion of 1040CS in 1 M HCl solution in the lack and attendance of unused ETC drug at 25 °C. Table 3 summarizes the percentage inhibition efficiency (% IE) and corrosion rate (k_corr_) values at temperature ranges 298, 303, 308, 313, and 318 K.

**Table 3:** Corrosion rate (k_corr_) and %IE data gotten from WL tests for 1040CS in 1 M HCl in the lack and attendance of altered doses of unused ETC at 25 °C.

Temp., K	[Inh] M	k_corr_ (mg cm^− 2^ min^− 1^)	$\:{\uptheta\:}$	% IE
25	0.0	0.6711	–	–
	50	0.1276	0.810	81.0
	100	0.1152	0.828	82.8
	150	0.1043	0.845	84.5
	200	0.0898	0.866	86.6
	250	0.0753	0.888	88.8
	300	0.0575	0.914	91.4
30	0.0	0.7781	–	–
	50	0.1804	0.768	76.8
	100	0.1649	0.788	78.8
	150	0.1502	0.807	80.7
	200	0.1302	0.833	83.3
	250	0.1111	0.857	85.7
	300	0.0935	0.880	88.0
35	0.0	0.9928	–	–
	50	0.2845	0.713	71.3
	100	0.2634	0.735	73.5
	150	0.2344	0.764	76.4
	200	0.2186	0.780	78
	250	0.1903	0.808	80.8
	300	0.1603	0.839	83.9
40	0.0	1.1876	–	–
	50	0.4459	0.625	62.5
	100	0.4322	0.636	63.6
	150	0.3929	0.669	66.9
	200	0.3365	0.717	71.7
	250	0.3032	0.745	74.5
	300	0.2785	0.766	76.6
45	0.0	1.3188	–	–
	50	0.6009	0.544	54.4
	100	0.5565	0.578	57.8
	150	0.5052	0.617	61.7
	200	0.4521	0.657	65.7
	250	0.3988	0.698	69.8
	300	0.3443	0.739	73.9

### Temperature effect

**Fig. 3:**
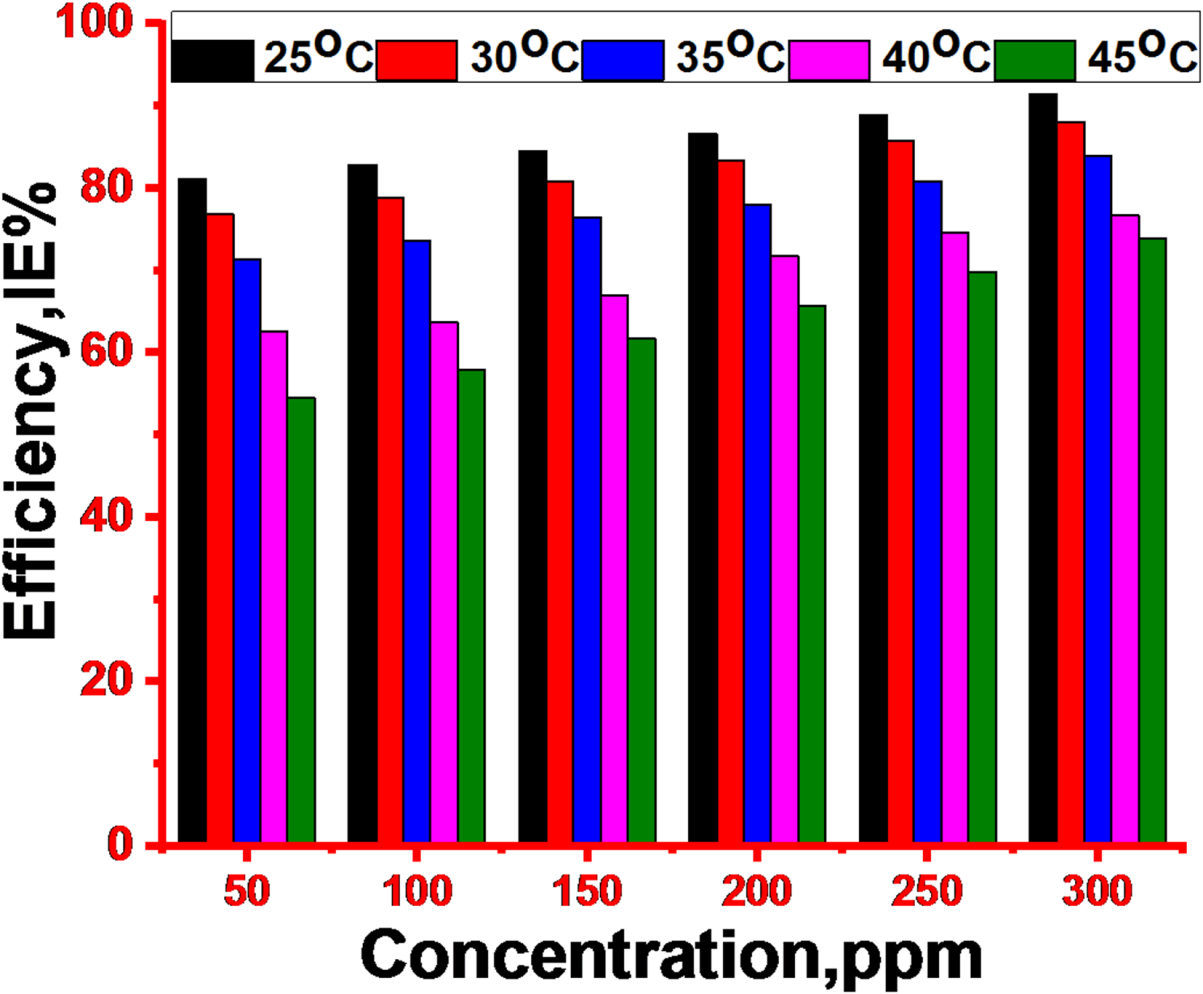
Effect of doses on the %IE of unused ETC at altered doses.

The WL method was used to investigate the effect of temperature on the corrosion characteristics of 1040CS by adding unused ETC drug. Additionally, experiments on WL were performed to examine the effect of temperature on the anticorrosion impact of the optimal concentration (300 ppm). Corrosion rates on the 1040CS sample increased with temperature in inhibited and uninhibited solutions. However, rates in inhibited solutions were significantly lower than in the acid without inhibitors. Table 3 shows the corrosion behaviour data of 1040CS in 1 M HCl with varying amounts of unused ETC for 120 minutes at temperatures between 25 and 45°C. While inhibitors suppressed corrosion at all temperatures, higher temperatures reduced their effectiveness. Inspection of this Table revealed that the k_corr_ of 1040CS rises with rising temperature. However, as the temperature rose, the percentage of unused ETC dropped (Fig. 3). This implied that some of the drug molecules that had been adsorbed would desorb from the metal surface at higher temperatures. Such behaviour shows that the drug was physically adsorbed on the metal surface”.

### Kinetic-thermodynamic corrosion parameters

**Fig. 4:**
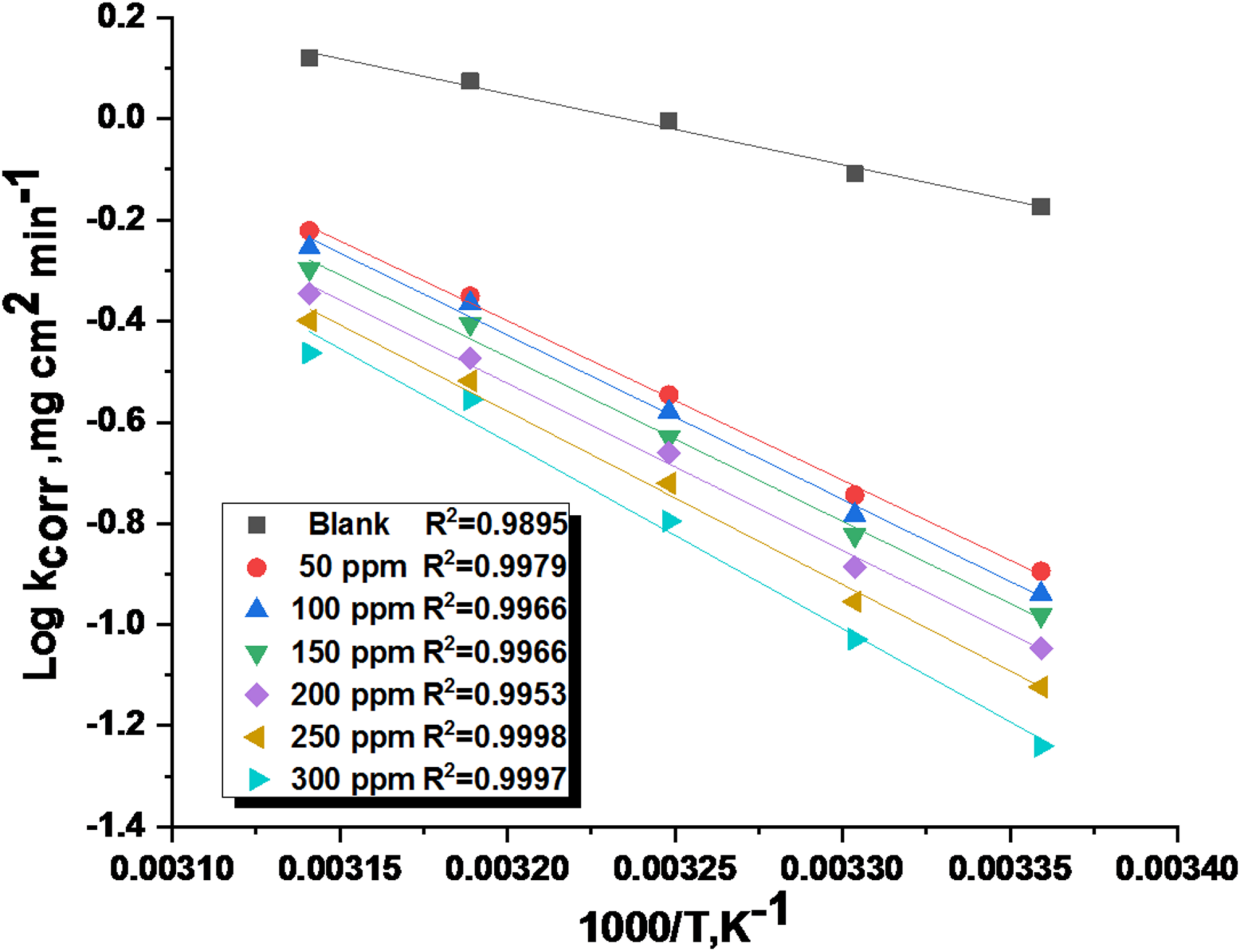
Arrhenius bends for 1040CS corrosion rates following a 120-minute dipping in 1 M HCl in the presence and absence of various ETC concentrations.

**Fig. 5:**
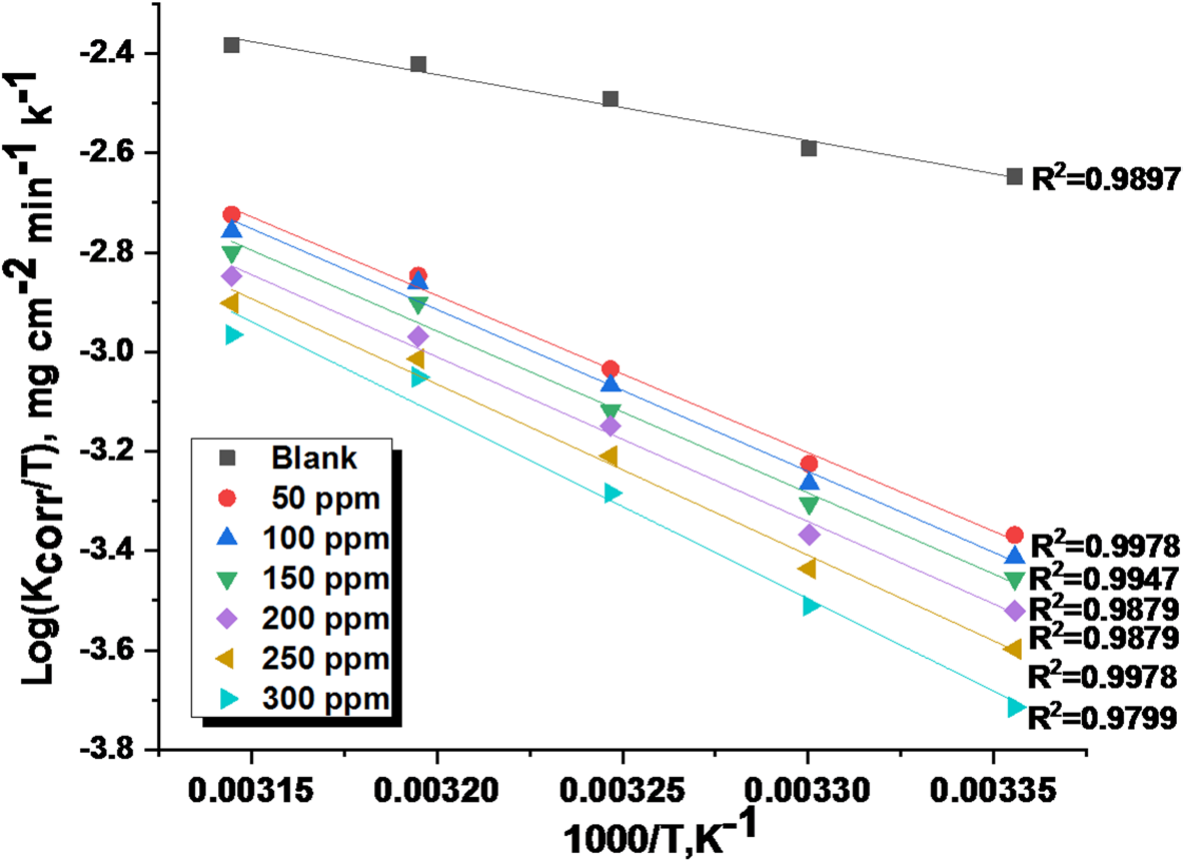
Transition-state for 1040CS corrosion rates in one molar HCl attendance and lack altered ETC concentrations.

For 1040CS corrosion in 1 M HCl solutions, the Arrhenius-type equation was used to calculate the apparent activation energy Ea*, the entropy of activation $\:\varDelta\:$S*, and the enthalpy of activation $\:\varDelta\:$H* in the presence and absence of altered doses of the studied ETC compound at 25–45 °C^44^.

5 $$\:\text{l}\text{o}\text{g}\:{\text{k}}_{\text{c}\text{o}\text{r}\text{r}}=\left(\frac{-{\text{E}}_{\text{a}}^{\text{*}}}{2.303\text{R}\text{T}}\right)+\:\text{l}\text{o}\text{g}\:\text{A}$$

where the Arrhenius pre-exponential component is denoted by (A), the values of Ea* were determined from plotting of log k_corr_ vs. 1000/T, which produced straight lines with a slope equal to − Ea* /2.303 RT, as illustrated in Fig. 4. From this slope, the Ea* values were computed. The transition-state equation was utilized for the intermediate complex to determine $\:\varDelta\:$H* and $\:\varDelta\:$S*^48^. And the equation of transition- state:

6 $$\:\frac{\text{l}\text{o}\text{g}{\text{k}}_{\text{c}\text{o}\text{r}\text{r}}}{\text{T}}=\text{log}\left(\frac{\text{R}}{\text{N}\text{h}}\right)+\frac{\varDelta\:{\text{S}}^{\text{*}}}{2.303\text{R}}-\frac{\varDelta\:{\text{H}}^{\text{*}}}{2.303\text{R}\text{T}}$$

where N is Avogadro’s number, and h is the Planck constant, The values of $\:\varDelta\:$H* and $\:\varDelta\:$S* were determined using a plot of Log (k_corr_/T) vs. 1000/T, as shown in Fig. 5, which has a slope equal to ( $\:\varDelta\:$H*/2.303R) as well as an intercept equals to [log (R/Nh) + ($\:\varDelta\:$S*/2.303R)]. In Table 4, all approximated kinetic-thermodynamic parameters were estimated.

Since the Ea* value increases as the concentration of the expired drug rises, we deduce that these medications create an energy barrier that increases as the drug concentrations do with the corrosion reaction. Higher energy barriers in inhibited solutions throughout the corrosion process are associated with physical adsorption^49^. The rise in ΔH* in the existence of the Unused ETC indicates that adding ETC to the acid solution raises the height of the energy barrier of the corrosion reaction to a certain degree, depending on the kind and dosage of the Unused ETC. This kind of behaviour could be attributed to an energy barrier, which is present in the corrosion process.

When drugs are present and absent, the activation entropy ($\:\varDelta\:$S*) is negative. The ΔS* values for the inhibited solution are less negative than those for the uninhibited, as the rational probability attributed to desorption of H_2_O from the CS surface. This demonstrates that, rather than representing dissociation in the rate-determining phase, the activated complex represents an association. In other words, a decrease in disordering occurs when reactants are transferred to the activated complex^50^.

**Table 4:** Activation parameters of 1040CS dissolution existence and lack various doses of unused ETC in 1 M HCl.

Conc.,	Activation parameters		
ppm	E_a_*	Δ Η^∗^	−ΔS^*^
	kJ mol^−1^	kJ mol^−1^	J mol^−1^ K^−1^
Blank	26.8	24.2	162.8
50	60.3	57.7	159.1
100	61.1	58.4	153.9
150	62.2	59.5	154.7
200	63.1	60.4	152.3
250	65.5	63.1	145.4
300	70.6	68.3	129.6

### Adsorption isotherms

**Fig. 6:**
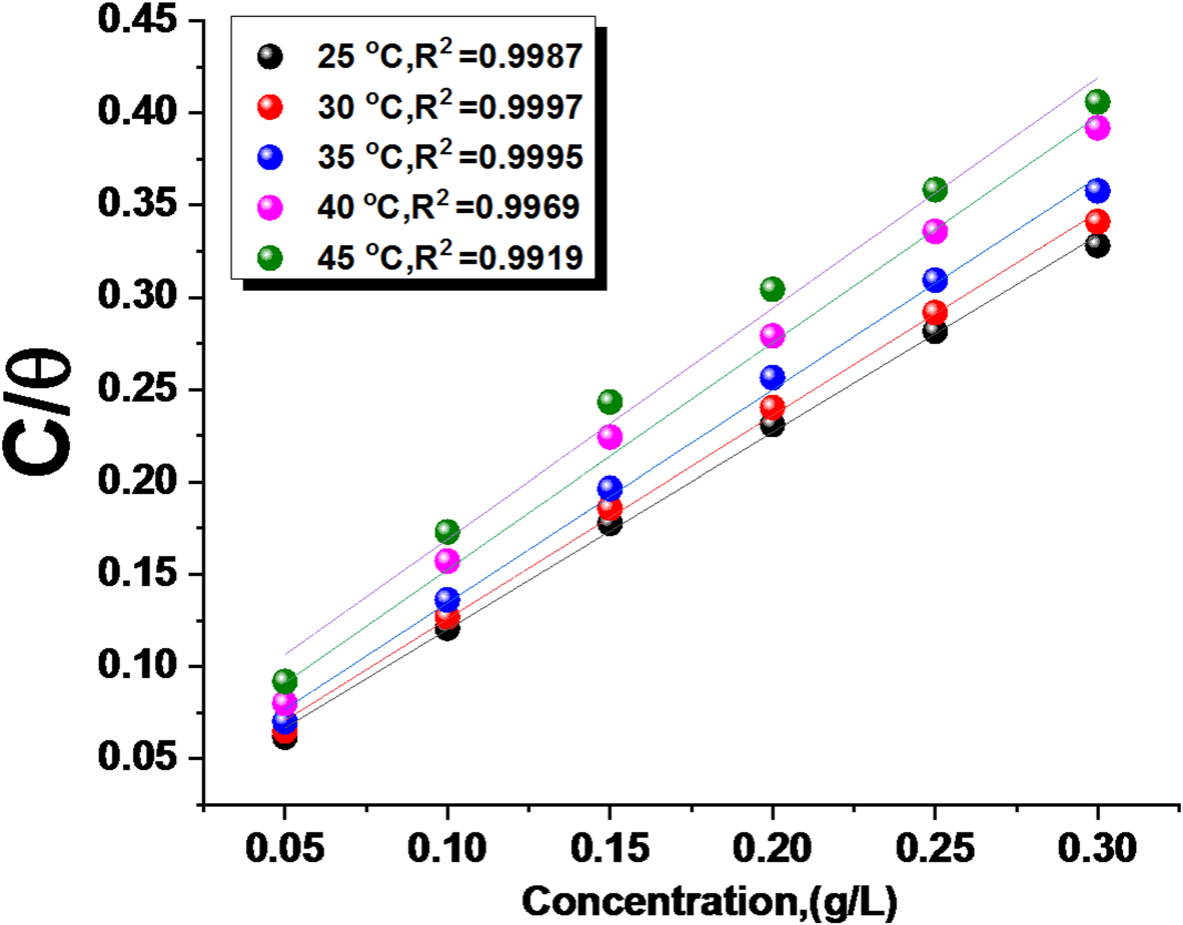
Langmuir adsorption of ETC on 1040CS surface in 1 M HCl at different temperatures.

Corrosion inhibition has been considered to begin with the adsorption phenomenon, in which inhibitor molecules tend to adsorb on the corroding surface layer. Several models, comprising Langmuir, Flory–Huggins, Freundlich, Frumkin, Temkin, and kinetic, were used to study the adsorption isotherms of ETC drug. This study examined the most suitable adsorption mode using the conventional weight loss method to find the extent of surface coverage filled by ETC molecule *(θ)*. Several mathematical relationships for the adsorption isotherms have been proposed to fit the experimental data of the present work. The Langmuir model with high fitting (R^2^ ranging between 0.9997 and 0.9919) was put forth (Fig. 6), and its applicability was evaluated using Eq. (7)^51^.

7 $${\text{C}}/{\text{q}}\,=\,{\text{1}}/{{\text{K}}_{{\text{ads}}}}+{\text{ C}}$$

The $\:{\varvec{K}}_{\varvec{a}\varvec{d}\varvec{s}}$ constant was computed from the intercept of the Langmuir plot, and $\:{\text{C}}_{\text{i}\text{n}\text{h}}$ represents the molecular weight of the ETC drug. According to the following thermodynamic equation, the free energy of adsorption ($\:{\varDelta\:\varvec{G}}_{\varvec{a}\varvec{d}\varvec{s}}^{0}$) is determined by using the $\:{\varvec{K}}_{\varvec{a}\varvec{d}\varvec{s}}$ constant:

8 $${K_{ads}}= \left( {1/55.5} \right) exp (D{G^\circ}_{{ads}}/ RT)$$

The ∆G°_ads_ data at all temperatures are documented in Table 5. The (∆H°_ads_.) was calculated agreeing to the Van’t Hoff Eq. 

9 $$\:{log}{k}_{ads}\:=\left(\frac{{-\varDelta\:H}_{ads}^{^\circ\:}}{2.303RT}\right)\:+constant$$

**Fig. 7:**
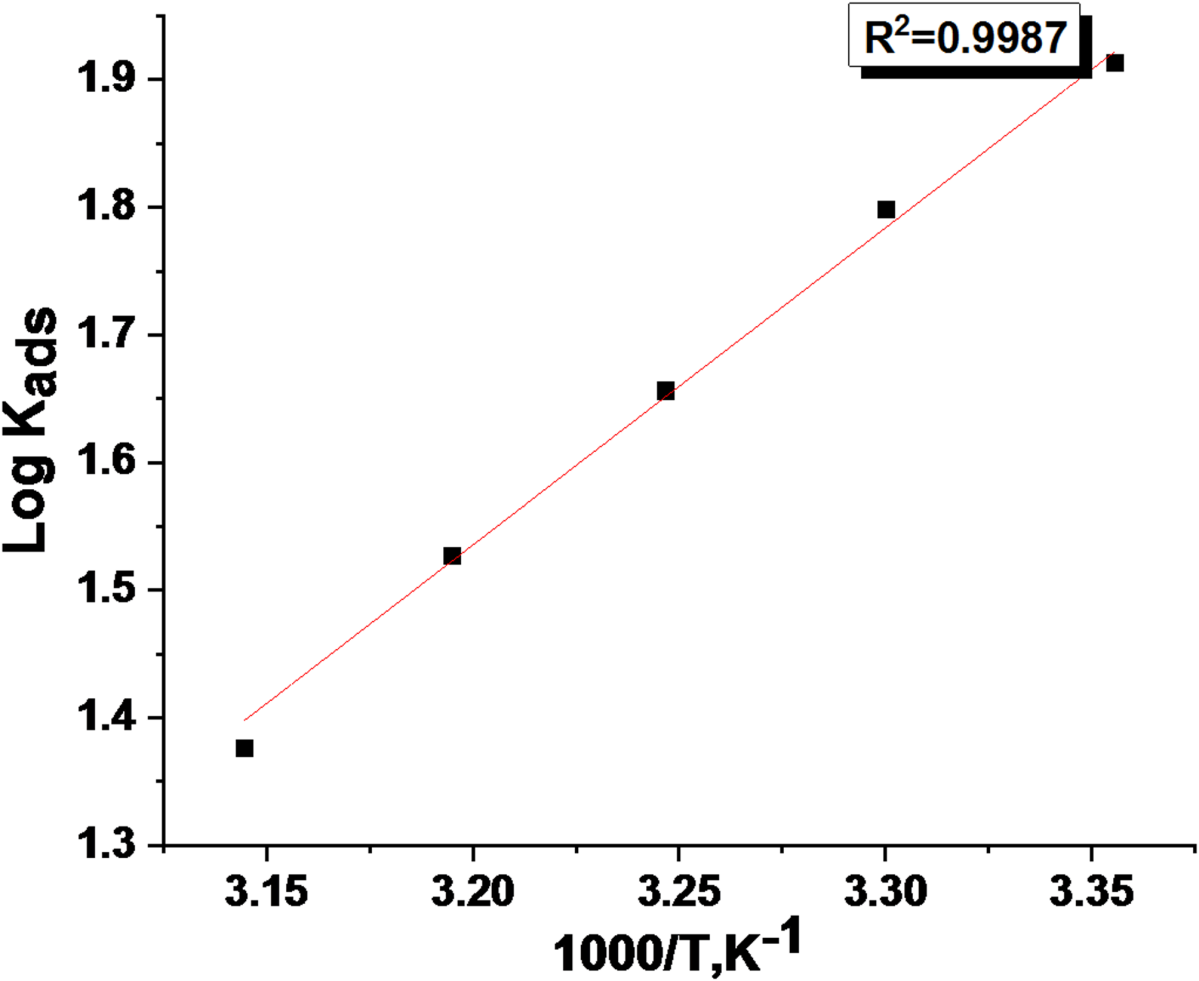
Log K_ads_ vs. 1000/T plots for examined ETC inhibitor at various temperatures.

Drawing (log K_ads_) vs. (1/T) gives a straight line as demonstrated in Fig. 7, the slope = (− ∆H°_ads_ /2.303R), from this slope; the ∆H°_ads_ data was computed and is displayed in Table 5. Then, by applying the following equation:

10 $$\Delta {G^\circ}_{{ads}}=\Delta {H^\circ}_{{ads.}} - T\Delta {S^\circ}_{{ads}}$$.

The adsorption characteristics for the derived Unused ETC medication are displayed in Table 5. “The table data confirmed the spontaneous adsorption of Unused ETC drug on the 1040CS surface, through the negative data obtained ΔG°_ads_, whose negative value lowered with higher temperature, which confirms that the adsorbed layer is more stable at lower temperatures. The decrease in K_ads_ values with increasing temperature further supports this, indicating weaker adsorption at higher temperatures. The magnitude of ΔG°_ads_ can provide information about the adsorption mechanism”. Values up to − 20 kJ/mol are characteristic of physisorption, involving electrostatic interactions between charged species. More negative values, below − 40 kJ/mol., indicate chemisorption, involving coordination interactions among the inhibitor and the metal surface. The results obtained from free energy confirm that the type of adsorption incident is physical adsorption and not chemical, as it is known that ΔG°_ads_ values when they are less than − 20 kJ/mol are physical adsorption; according to these results established that the adsorption is physisorption. The negative ΔH°_ads_ values in Table 5 indicate an exothermic adsorption process, supporting both physisorption and chemisorption of ETC on the 1040CS surface, but the value governs the kind of adsorption. ∆H°_ads_ value less than 40 kJ/mol refer to the physisorption procedure. The positive ΔS⁰_ads_ values indicate an increase in entropy at the metal-inhibitor interface, which may be associated with desorption of H_2_O molecules from the metal surface during physisorption^52^.

**Table 5:** Parameters of Langmuir adsorption of unused ETC on the 1040CS surface at change temperatures.

Temp ºC	K_ads_	−ΔG°_ads_, kJ mol^−1^	−ΔHº_ads_ kJ mol^−1^	ΔSº_ads_ J mol^−1^ K^−1^
25	75	20.7	47.5	69.1
30	96	20.5		67.8
35	52	20.4		66.1
40	33	19.6		62.5
45	22	18.9		59.2

### Electrochemical tests

#### PDP measurements

**Fig. 8:**
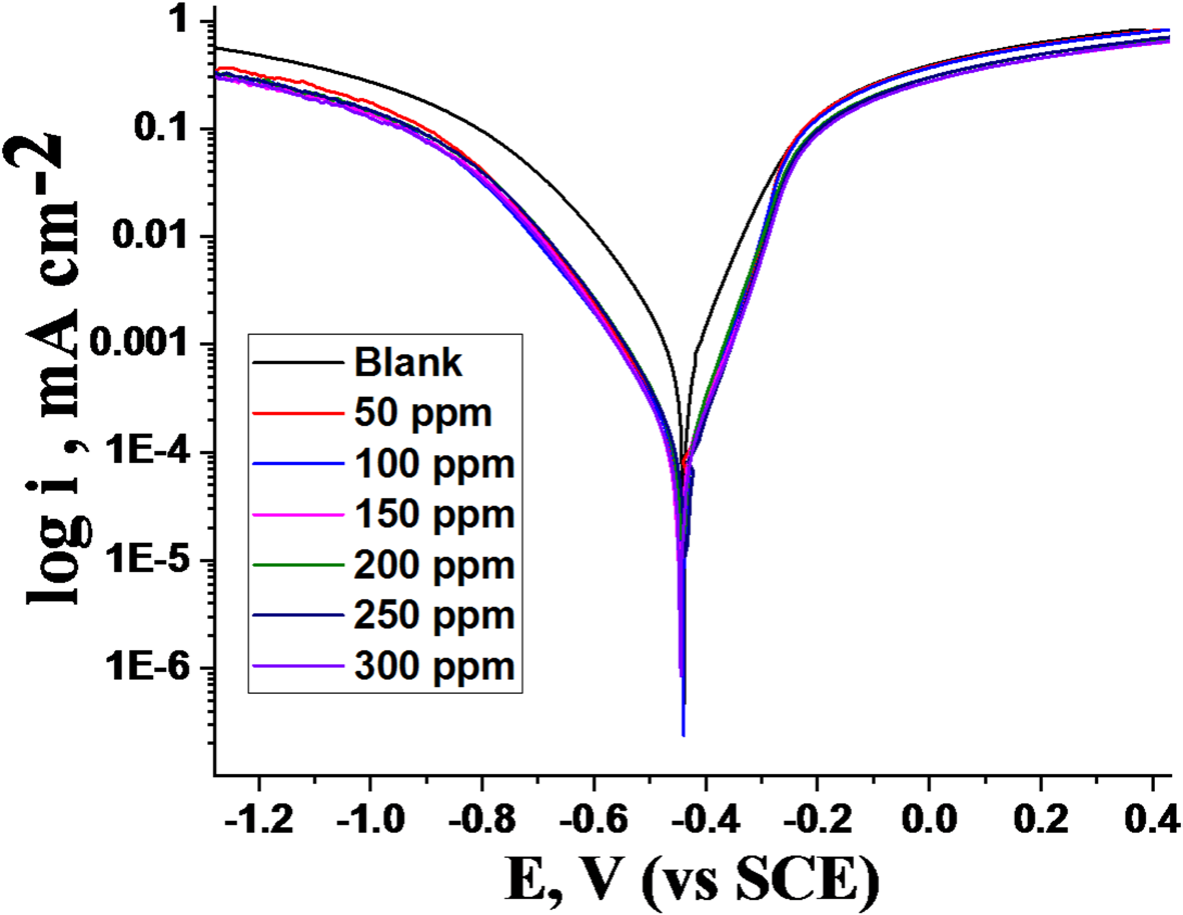
Potentiodynamic polarization curves of 1040CS dissolution with and without altered doses of unused ETC at 25 °C.

The cathodic and anodic polarization bends for 1040CS in 1 M HCl in uninhibited and inhibited solutions of unused ETC at 25 °C are shown in Fig. 8. By extrapolating the linear portions of the anodic and cathodic Tafel regions to their intersection point, we can determine the corrosion current density (i_corr_). This parameter is vital for quantifying the rate of metal dissolution and evaluating the efficacy of corrosion inhibitors. The electrochemical parameters, including anodic and cathodic Tafel slopes, were extracted from the polarization curves (Table 6). The presence of the ETC drug led to a significant decrease in corrosion current density (i_corr_), signifying a reduction in the overall corrosion rate. This decrease, coupled with rise in IE %, can be attributed to the adsorption of ETC molecule onto the 1040CS surface^53–55^. This adsorption process forms a protective film that acts as a barrier, hindering both the metal’s anodic dissolution and the hydrogen ions’ cathodic reduction. According to the cathodic and anodic polarisation curves, the ETC drug reduces current densities and modifies E_corr_ values slightly. Since the shift of the E_corr_ potential at the various inhibitor doses is less than 85 mV compared to the corrosion potential without ETC, it confirms a mixed-type inhibitor. The corrosion potential (E_corr_) shifted slightly towards the cathodic region, with a maximum displacement of 13 mV, suggesting that the ETC drug acts as a mixed-type inhibitor. These results corroborate those of the literature^56^. Changes in anodic (β_a_) and cathodic (β_c_) Tafel slopes indicate that ETC drug adsorption modifies both anodic dissolution and cathodic hydrogen evolution mechanisms. This outcome data suggests that the mechanism of inhibition remains unchanged when the unused ETC is present or not^57,58^.

**Table 6:** Effect of unused ETC doses on corrosion parameters of 1040CS in 1 M HCl at 25 °C.

Conc, ppm	i_corr_. µA cm^− 2^	− E_corr_. mV vs. SCE	β_a_ mV dec^− 1^	β_c_ mV dec^− 1^	k_corr_ mpy	Θ	% IE
0.0	994	439	100	141	353	–	–
50	142	443	96	131	64	0.857	85.7
100	133	440	93	129	60	0.866	86.6
150	120	448	92	127	54	0.880	88.0
200	130	441	89	124	50	0.869	86.9
250	120	435	88	123	48	0.880	88.0
300	93	445	87	122	42	0.907	90.7

#### EIS measurements

**Fig. 9:**
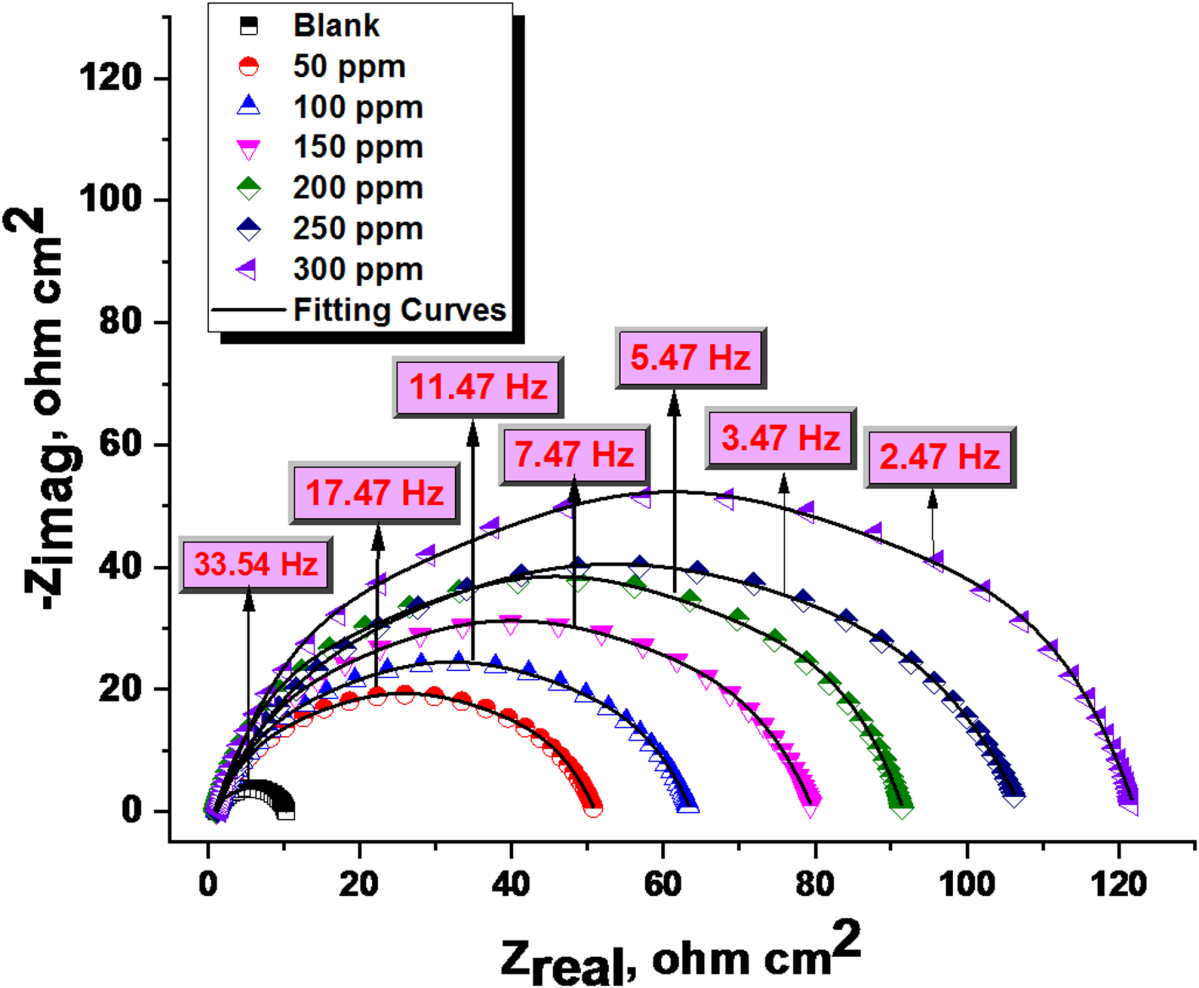
Nyquist curves of EIS spectra for the dissolution of 1040CS in 1 M HCl existence and lack altered doses of unused ETC at 25 °C.

**Fig. 10:**
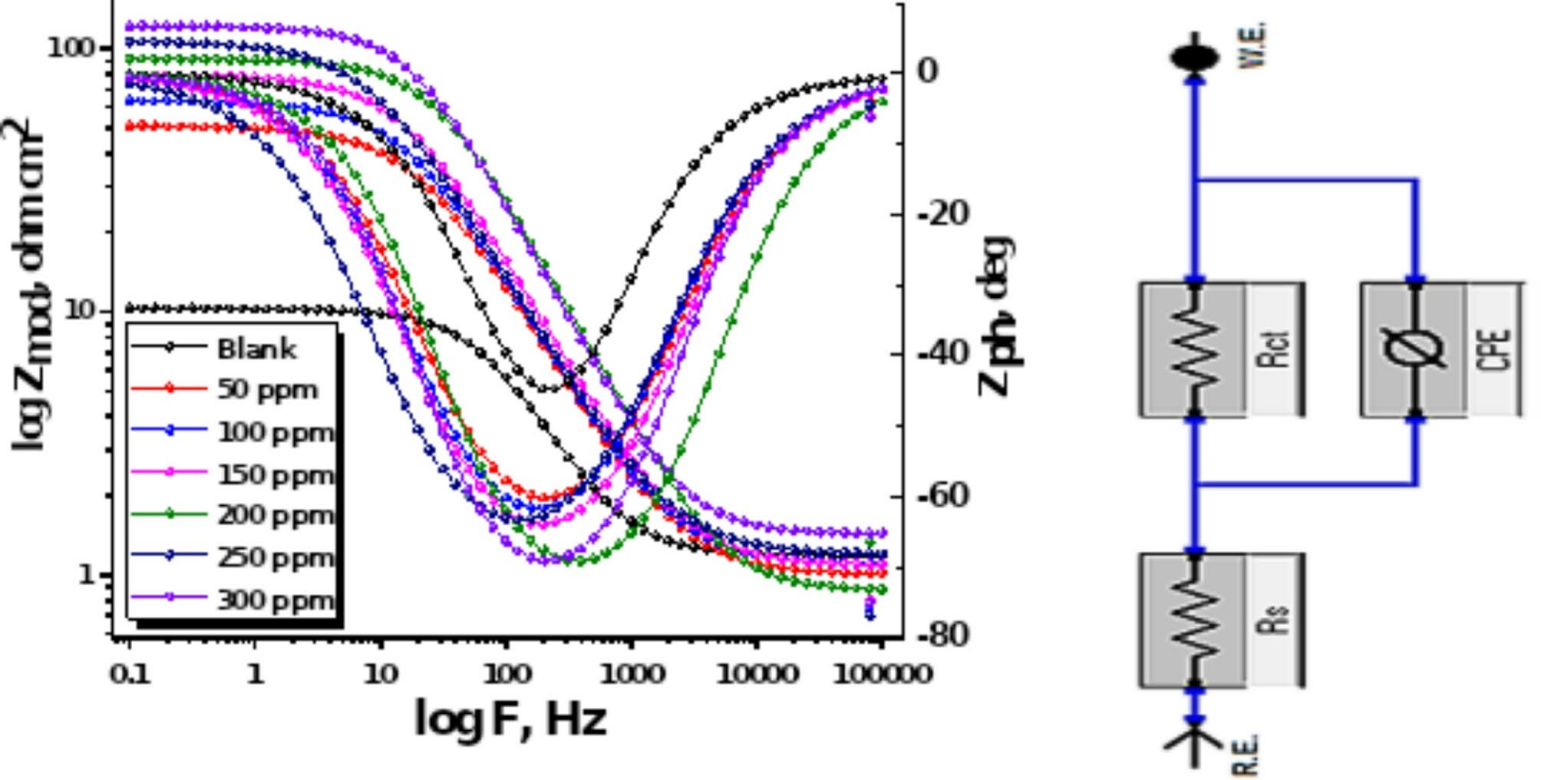
Bode plots for EIS spectra for the dissolution of 1040CS in 1 M HCl in the lack and existence of altered doses of Unused ETC at 25 °C and equivalent circuit model utilized to fit the EIS spectra.

In Fig. 9, the Nyquist curve for 1040CS in 1 M HCl is displayed with and without modified dosages of unused ETC at 25 °C. This semicircular diagram shows that a charge transfer procedure is primarily responsible for controlling unused ETC corrosion. Figure 10 displays the Bode plot and equivalent circuit model used to fit the impedance spectra for the 1040CS. The high-frequency limit is associated with the electrolyte resistance R_s_, and the low-frequency limit is the sum of (R_s_ + R_ct_), where R_ct_ is the first approximation based on the oxide film’s electrolytic conductance as well as the polarisation resistance of the dissolution and passivation process.

Various impedance parameters such as charge transfer resistance (R_ct_), double layer capacitance (C_dl_), and (% IE) were calculated and are given in (Table 7). The data obtained displayed that the charge transfer resistance (Rct) values rise. The data of layer (C_dl_) capacitance double lowered with improving the dose of the unused ETC, which was accompanied by a rising (% IE) due to the adsorption of this ETC molecule on the electrode surface, leading to a film formation on 1040CS surface. The values of C_dl_ were assessed by exploiting the following formula:

11 $$\:{\varvec{C}}_{\varvec{d}\varvec{l}}=\:\frac{1}{2\varvec{\pi\:}{\varvec{f}}_{\varvec{m}\varvec{a}\varvec{x}}{\varvec{R}}_{\varvec{c}\varvec{t}}}$$

where f_max_ is the frequency at the highest altitude of the half circle, a change in inhibitor concentration between 50 ppm and 300 ppm, respectively, increases the charge transfer resistance and decreases the capacity of the electric double layer capacitor. Further, the double-layer capacitance decreases significantly. According to this explanation, the dielectric constant at the double layer has decreased, and/or its electrical thickness has increased. ETC drug adsorb on the CS surface in 1.0 M HCl, replacing water molecules and decreasing C_dl_^59^. Small chi-squared (*χ*^2^) values (Table 7) indicate strong agreement between fitted results and experimental data, also shows that (n) value varies directly with drug dose, “whereas Y_o_ (the constant phase element introduced to compensate for the frequency depression phenomenon) does not. (n) value is a measure of surface roughness^60,61^and its increase in this study could mean a decrease in the heterogeneity of the working electrode surface due to drug adsorption”.

**Table 7:** EIS data for dissolution 1040CS in 1 M HCl existence and lack altered doses of unused ETC at 25 °C.

Conc, ppm	Y_o, µ Ω_^-1^_s_^*n*^_cm_^-2^_×10_^-6^	*n*	*R*_*p*_, Ω cm^2^	C_dl_, µFcm^− 2^	θ	% IE	Goodness of fit (χ^2^)
0.0	532	0.821	8	162	–	–	15.47 × 10^− 4^
50	357	0.833	50	159	0.840	84.0	16.71 × 10^− 4^
100	326	0.840	62	155	0.871	87.1	21.24 × 10^− 4^
150	260	0.849	79	130	0.899	89.9	18.57 × 10^− 4^
200	116	0.887	91	65	0.912	91.2	16.21 × 10^− 4^
250	110	0.889	105	63	0.924	92.4	13.23 × 10^− 4^
300	97	0.901	120	59	0.933	93.3	16.88 × 10^− 4^

### Surface examinations

#### SEM tests

**Fig. 11:**
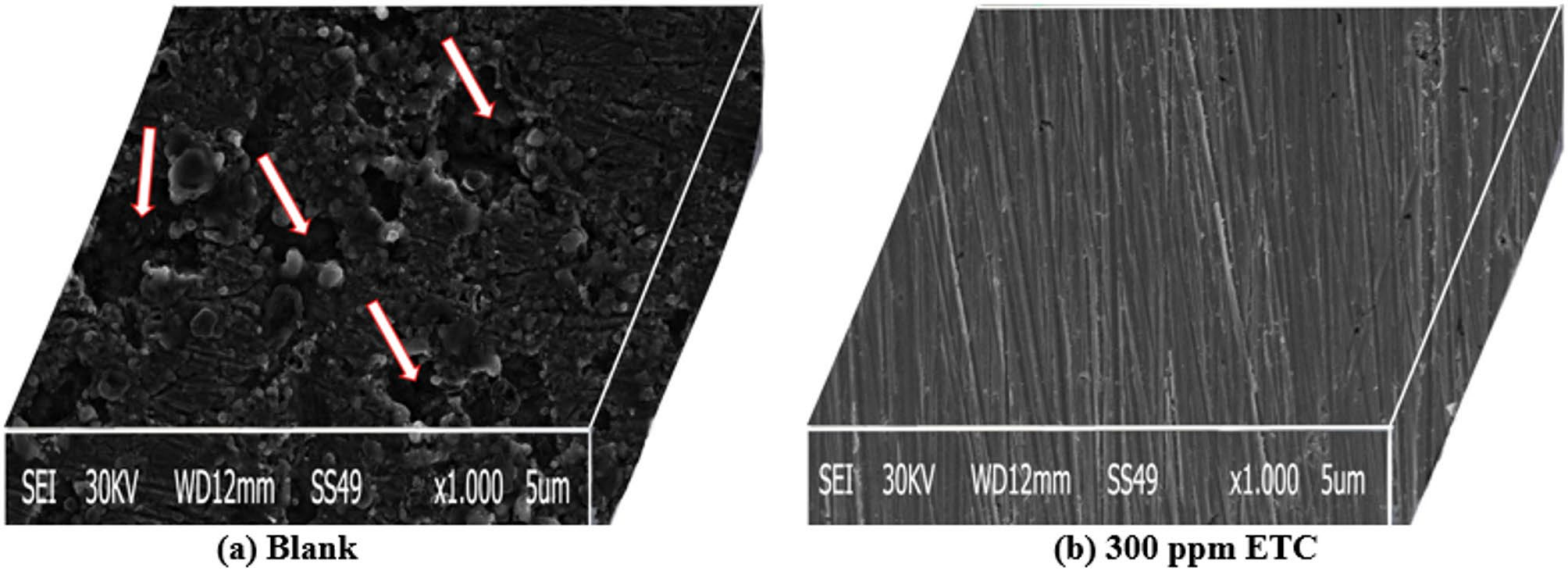
SEM image of 1040CS surface, (**a**) 1040CS surface after 24 h of soaking in 1.0 M HCl, (**b**) CS surface after 24 h of soaking in 1.0 M HCl in the presence of 300 ppm ETC inhibitor.

SEM examination of the electrode surface showed that a protective inhibitory surface coating had formed^62^. The micrographic of the 1040CS sample acquired following 24 h of immersion in 1 M HCl is shown in Fig. 11. The CS surface is severely attacked by corrosion (Fig. 11a). The surface of CS following exposure to 1 M HCl solution containing 300 ppm of ETC drug inhibitor is shown in Fig. 11b. It is necessary to highlight that the morphology of 1040CS surfaces changes significantly and becomes smoother in the presence of an ETC inhibitor. Across the whole surface of the CS, a haphazard-distributed layer is created. This layer means that inhibitor molecules interact with the CS surface’s reaction sites, reducing contact between the metal and the aggressive medium and leading to corrosion rate breakdown. Alternatively, it could mean that the CS surface’s compounds are adsorbing onto the passive film and incorporating into it to block the active site on the CS surfaces.

#### AFM analysis

**Fig. 12:**
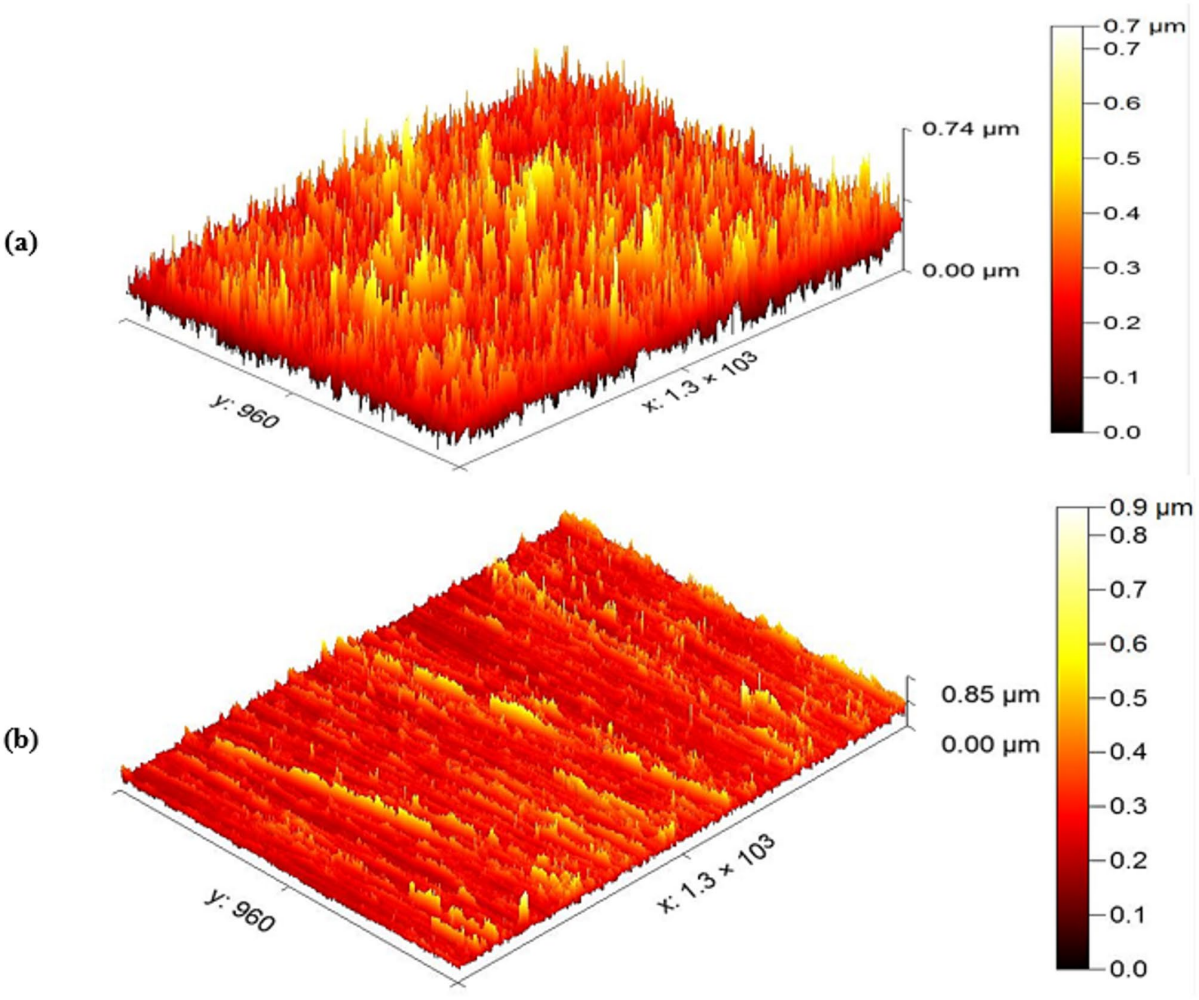
AFM images for exposed 1040CS in blank solution (**a**), exposed 1040CS in 1 M HCl containing 300 ppm ETC inhibitor (**b**) after 24 h dipping at 25 °C.

One of the most crucial methods for researching surface morphology is AFM. AFM analyses surface roughness and creates a three-dimensional picture of CS surface^63^. CS samples are confronted in 1 M HCl for only three hours at room temperatures, both with and without 300 ppm of inhibitors. The 3D pictures of the 1040CS surface are shown in Fig. 12.

The roughness values of 1040CS surface in 1 M HCl are exclusively high (647 nm) (Fig. 12a), while 1040CS surface roughness levels are lower when inhibitors are present than when they are not (105 nm) (Fig. 12b). Because an adsorbed layer has formed, the electrode surface is virtually free of corrosion. At the electrode surface, an inhibitory coating serves as protection.

#### FT-IR analysis

**Fig. 13:**
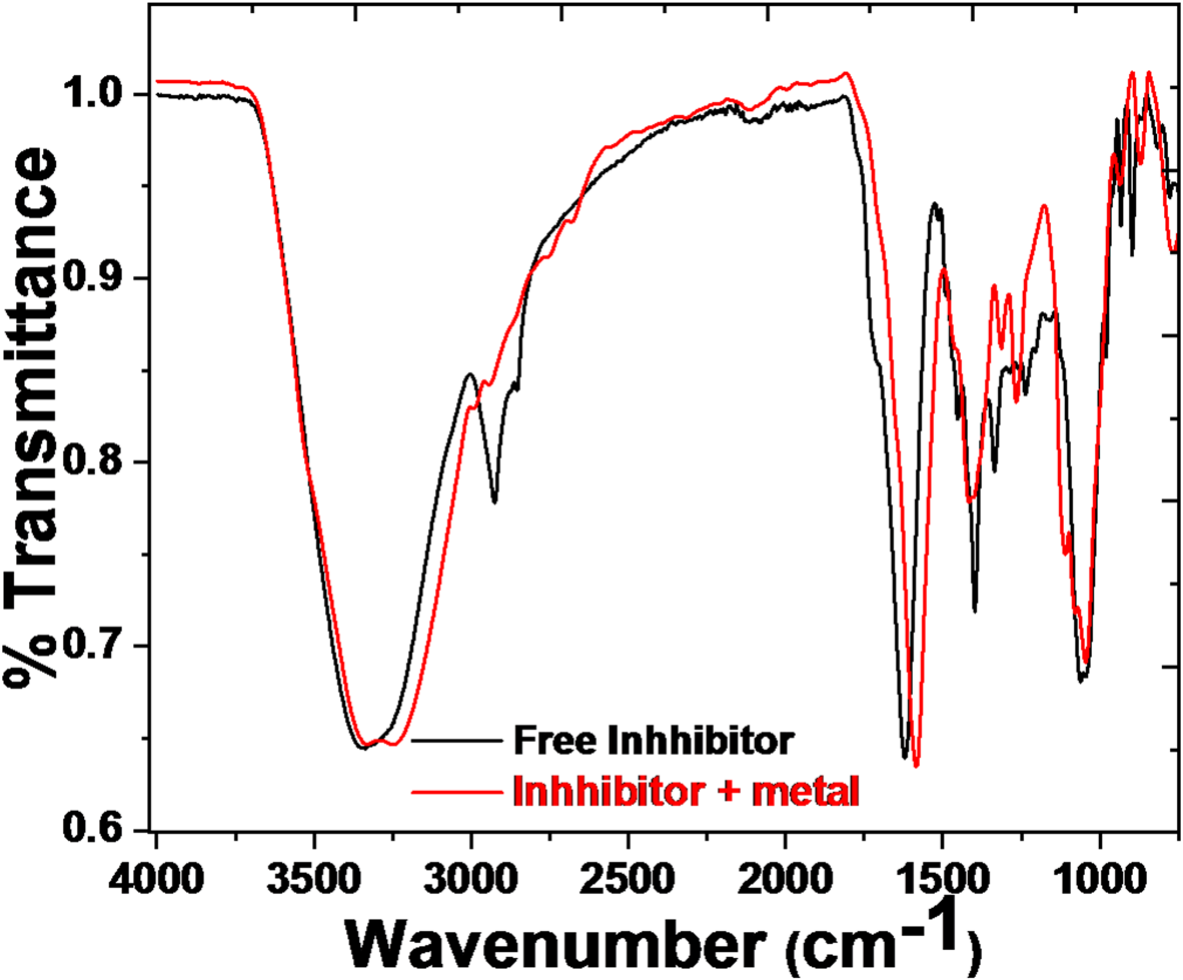
IR spectrum of pure Unused ETC drug at 25 °C.

FT-IR spectroscopy presents exciting capabilities such as excessive signal-to-noise ratio, excessive sensitivity and selectivity, accuracy, mechanical simplicity, short analysis time, and small sample required for the analysis. Employing FT-IR analysis for the adsorbed layer on the 1040CS surface, the function groups of the inhibitors are identified. The function groups of the drug were represented by specific peaks that emerged in the infrared spectra using this method. Figure 13 display the investigated inhibitors’ FT-IR spectra, which provide the drug fingerprint spectrum and the CS surface after being submerged in 1 M HCl plus the highest concentration of inhibitors. When these were compared, it was evident that the inhibitor’s stock solution fingerprint was present on the 1040CS surface, except for a few functional groups that were thought to result from the reaction with HCl. FT-IR can be used to analyze drugs since they contain a variety of organic molecule called inhibitors, which are adsorbed on the metal surface and prevent corrosion^64^.

### Quantum chemical parameters

**Fig. 14:**
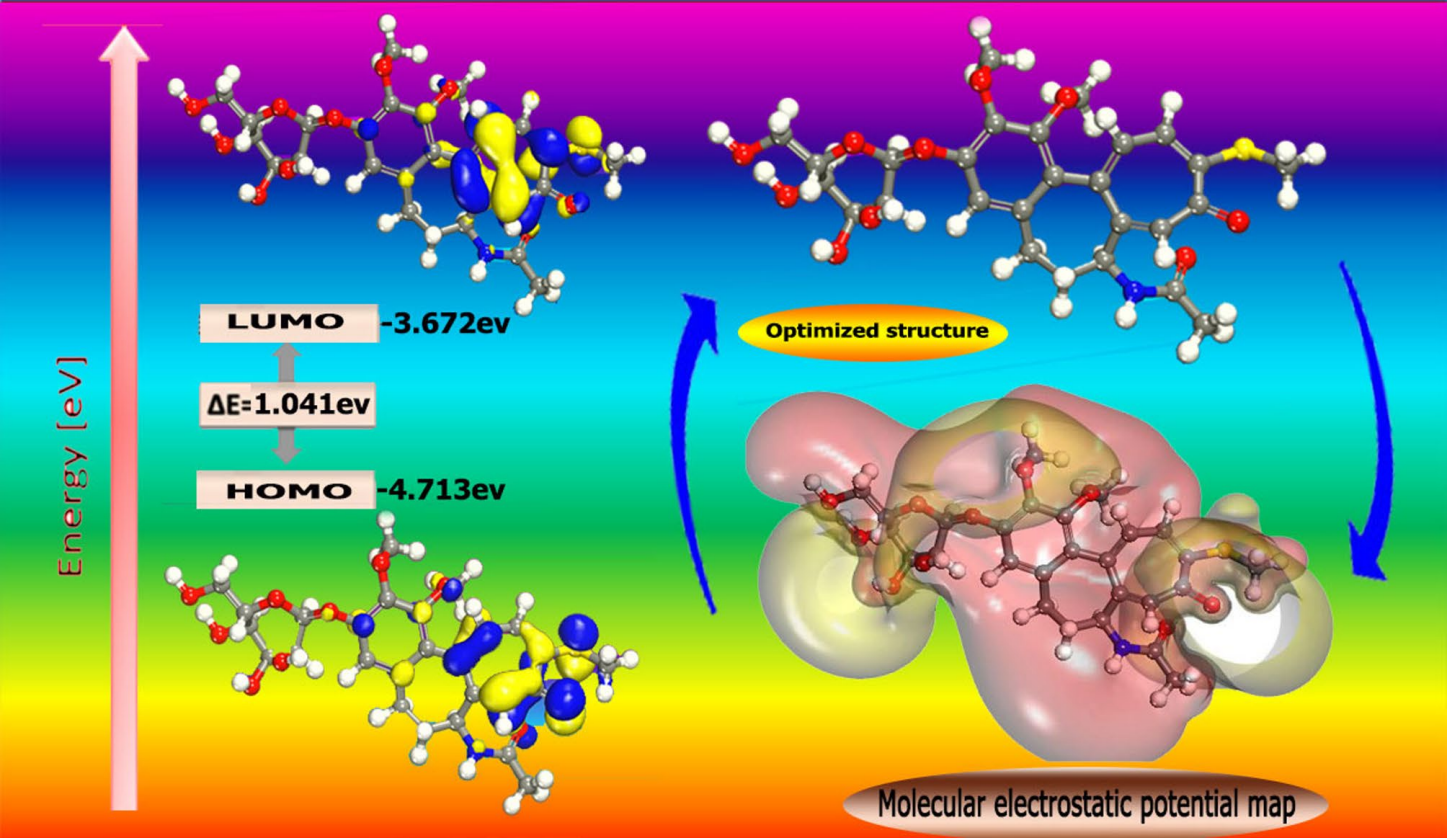
The frontier molecular orbital Unused ETC inhibitor (HOMO and LUMO) (Material Studio software (version 7.0) https://www.fullversiondl.com/accelrys-materials-studio-v7-0/).

Table 8 includes the lowest unoccupied molecular orbital (E_LUMO_), highest occupied molecular orbital (E_HOMO_), energy gap $\:(\varDelta\:E$), dipole moment ($\:\mu\:),\:$electron affinity (A), electronegativity (χ), ionization potential(I), softness (σ) and global hardness (η). Firstly, E_HOMO_ illustrates the ability of the inhibitor to donate electrons to the empty d-orbital, which is present on the CS surface. By increasing its value, the %IE will increase, and the adsorption process will be more facilitated and easier^65^. On the other hand, E_LUMO_ indicates the tendency of molecule to accept electrons. Reducing the value of E_LUMO_ will boost a molecule’s capacity to receive electrons (Fig. 14). The polarity of the examined ETC compound was determined using the (µ) calculation. It is acknowledged that the high µ data improves the adsorption affinity of unused ETC on the 1040CS surface. Since ∆E represents the energy required to remove an electron from the final occupied orbital, it was deemed desirable to be low. Lower energy gap values are associated with ETC molecule that are highly reactive and have good corrosion inhibition efficiency on metal surface. This equation is used to calculate it^66^:

12 $$\:\varDelta\:E={E}_{LUMO}-{E}_{HOMO}$$

Additionally, as it connects the ionization potential and the electron affinity, A and I are connected to the E_HOMO_ and the E_LUMO_. The absolute electronegativity, χ, and hardness, η, respectively, Equations following illustrate the calculation of the inhibitor molecule’s global softness, σ.

13 $$\:I=-{E}_{HOMO}$$

14 $$\:A=-{E}_{LUMO}$$

15 $$\:\chi\:=\frac{I+A}{2}=-\frac{{E}_{HOMO}+{E}_{LUMO}}{2}$$

16 $$\:\eta\:=\frac{I-A}{2}=\frac{{E}_{LUMO}-{E}_{HOMO}}{2}=\frac{1}{2}{\varDelta\:E}_{L-H}$$

17 $$\:\sigma\:=\frac{1}{\eta\:}$$.

**Table 8:** Parameter gotten from quantum for unused ETC drug.

Parameters (variable)	BMPP
E_HOMO_ (eV)	− 4.713
E_LUMO_ (eV)	− 3.672
∆E, (eV) (E_L_–E_H_)	1.041
E_A_ (eV)	4.71
I_p_ (eV)	3.67
(eV)χ (electronegativity)	4.19
µ	− 4.19
η, eV	0.52
σ, eV	1.92
ω, eV	16.88
ΔΝ	2.70
Δ*E*_backdonation_	− 0.13
Dipole moment (debye)	6.205
Molecular surface area, Å2	526.73

“The results show that the number of electrons transferred (ΔN) of the inhibitor molecules studied is less than 3.6. This suggests the ease of electron transfer from the inhibitor to the empty d orbital of the metal surface^67^”.

### Monte Carlo (MC) simulation

**Fig. 15:**
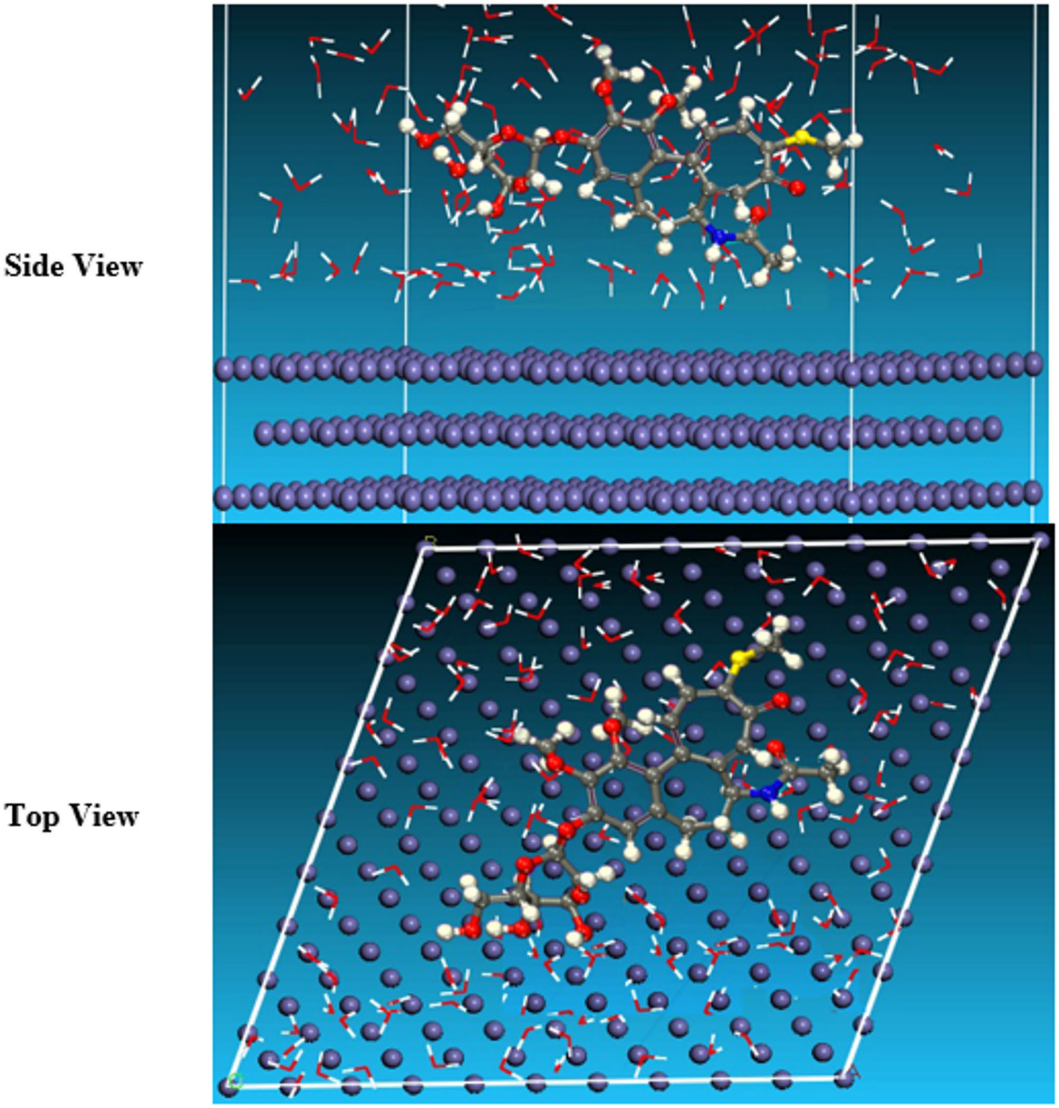
The most appropriate conformation for adsorption of the Unused ETC molecule on Fe (1 1 0) (Material Studio software (version 7.0) https://www.fullversiondl.com/accelrys-materials-studio-v7-0/).

The adsorption locator module’s side and top observations of the best adsorption formations for the unused ETC drug tested on the 1040CS surface are thus displayed in Fig. 15. Adsorption energy is characterized as declining energy when two materials are mixed during the adsorption process in which an electron, ion, or molecule (adsorbent) is bound to the solid surface. Table 9 presents the results of this approach. The E_total_ energy obtained for the ETC inhibitor is − 1460.814 kJ mol^− 1^, and E_ads_ due to the adsorption of ETC inhibitor onto the Fe(1 1 0) surface was − 1488.354 kJ mol^− 1^. The E_ads_ value proves that ETC inhibitor adsorbs on 1040CS substrates spontaneously^68^. Unused ETC has higher energy for adsorption, which predicts the heavy adsorption of Unused ETC on the hardened surface of 1040CS creating adsorbed stable layers which protecting the 1040CS from corrosion.

**Table 9:** Results and descriptors were measured using the Monte Carlo simulation for the adsorption of unused ETC molecule on iron (1 1 0).

Structures	Total energy	Adsorption Energy, kcal mol^−1^	Rigid adsorption energy, kcal mol^−1^	Deformation energy, kcal mol^−1^	Compound dE_ads_/dN, kcal mol^−1^	H_2_O dE_ads_/dNi, kcal mol^−1^
Fe (1 1 0)/ Unused ETC/H_2_O	− 1460.8	− 1488.4	− 1418.6	− 69.74	− 118.49	− 26.17

.

### Mechanism of corrosion Inhibition

**Fig. 16:**
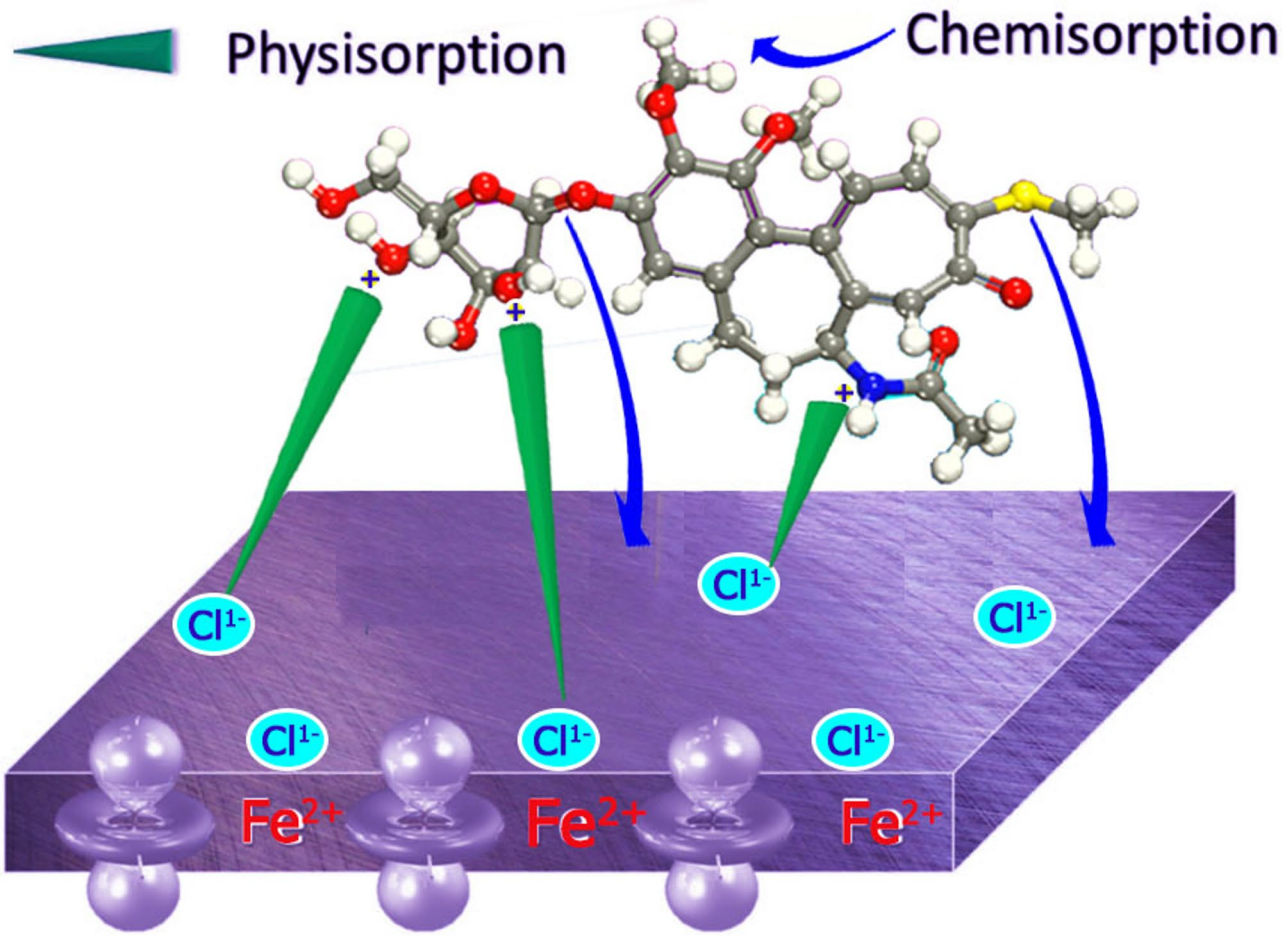
Mechanism of inhibition (Microsoft 365PowerPoint, https://www.microsoft.com/en-us/microsoft-365/powerpoint).

The inhibitor stops working or shows its effect until it binds to the 1040CS surface. This adsorption may be physical or chemical, so many parameters affect the adsorption process and degree of inhibition, such as metal surface and inhibitor charges, number of heteroatoms and aromatic ring, corrosive media composition, and electron density. Figure 16 shows the adsorption of drugs on 1040CS surface^69^. The inhibitors adsorbed chemically on the 1040CS surface in a special way. The adsorption occurred by transferring unshared electrons of heteroatoms and benzene rings to the empty d-orbitals of iron atom on the CS surface, forming a complex between Fe and inhibitor by coordination bonding. Figure 16 illustrates the adsorption mechanism of a particular ETC drug in HCl on the 1040CS surface under acidic conditions. Due to the aggressive acidic environment, the 1040CS surface oxidized rapidly and positively charged. This allowed the attraction of negatively charged chloride anions, resulting in a negative metallic surface. The negatively charged metal surface attracts the protonated ETC inhibitor molecule, causing it to adhere through electrostatic forces. This behavior leads to forming a protective coating layer on the 1040CS surface, which acts as a barrier to prevent its interaction with the corrosive environment. This layer protects the 1040CS surface from corrosion^70^.

## Conclusion

The studied ETC are effective inhibitors of 1040CS corrosion in 1 M HCl solution; nevertheless, their structures determine their effectiveness, and the kind and nature of the substituents in the inhibitor molecules determine how different they have affected their inhibitive efficiency. The concentration, temperature, and kind of metal and inhibitor all affect the inhibitor ability to adsorb. All measurements and surface analyses indicate that the ETC drug has remarkable inhibitory efficiency; at a concentration of 300 ppm and a temperature of 25 °C, the inhibition rate reached 91.3%. The inhibition of 1040CS in 1 M HCl solution was found to obey Langmuir adsorption isotherm. The thermodynamic parameters revealed that the adsorption of the ETC is of mixed type inhibitor. The results obtained from potentiodynamic polarization showed that ETC compound behave as mixed type inhibitor by inhibiting both anodic metal dissolution and cathodic hydrogen evolution reaction. The overall findings of this study contribute to the body of knowledge regarding the mechanism by which unused ETC prevents corrosion and its possible use as a protective agent on 1040CS in hydrochloric acid environments. The existence of the protective film on the surface of 1040CS was proved by AFM, SEM and FT-IR. Theoretical calculations align with experimental findings, showing that ETC inhibitor revelations greater inhibition efficiency. Results gained from all utilized techniques (chemical, electrochemical and quantum measurements) were in good agreement.

## Data Availability

Data is provided within the manuscript.
